# SP-8356, a Novel Inhibitor of CD147-Cyclophilin A Interactions, Reduces Plaque Progression and Stabilizes Vulnerable Plaques in apoE-Deficient Mice

**DOI:** 10.3390/ijms21010095

**Published:** 2019-12-21

**Authors:** Kisoo Pahk, Chanmin Joung, Hwa Young Song, Sungeun Kim, Won-Ki Kim

**Affiliations:** 1 Institute for Inflammation Control, Korea University, Seoul 02841, Korea; kisu99@korea.ac.kr (K.P.); joungchanmin@korea.ac.kr (C.J.); shy997@korea.ac.kr (H.Y.S.); 2 Department of Neuroscience, Korea University College of Medicine, Seoul 02841, Korea; 3 Department of Nuclear Medicine, Korea University Anam Hospital, Seoul 02841, Korea; seiong@korea.ac.kr

**Keywords:** atherosclerosis, plaque, plaque vulnerability, cluster of differentiation 147 (CD147), matrix metalloproteinase, matrix metalloproteinase-9 (MMP-9)

## Abstract

Interactions between CD147 and cyclophilin A (CypA) promote plaque rupture that causes atherosclerosis-related cardiovascular events, such as myocardial infarction and stroke. Here, we investigated whether SP-8356 ((1S,5R)-4-(3,4-dihydroxy-5-methoxystyryl)-6,6-dimethylbicyclo[3.1.1]hept-3-en-2-one), a novel drug, can exert therapeutic effects against plaque progression and instability through disruption of CD147-CypA interactions in apolipoprotein E-deficient (ApoE KO) mice. Immunocytochemistry and immunoprecipitation analyses were performed to assess the effects of SP-8356 on CD147-CypA interactions. Advanced plaques were induced in ApoE KO mice via partial ligation of the right carotid artery coupled with an atherogenic diet, and SP-8356 (50 mg/kg) orally administrated daily one day after carotid artery ligation for three weeks. The anti-atherosclerotic effect of SP-8356 was assessed using histological and molecular approaches. SP-8356 interfered with CD147-CypA interactions and attenuated matrix metalloproteinase-9 activation. Moreover, SP-8356 induced a decreased in atherosclerotic plaque size in ApoE KO mice and stabilized plaque vulnerability by reducing the necrotic lipid core, suppressing macrophage infiltration, and enhancing fibrous cap thickness through increasing the content of vascular smooth muscle cells. SP-8356 exerts remarkable anti-atherosclerotic effects by suppressing plaque development and improving plaque stability through inhibiting CD147-CypA interactions. Our novel findings support the potential utility of SP-8356 as a therapeutic agent for atherosclerotic plaque.

## 1. Introduction

Atherosclerosis is a major cause of mortality and morbidity worldwide associated with characteristic clinical symptoms, myocardial infarction, and stroke [1,2]. The high incidence of cardiovascular disease linked with atherosclerosis further imposes a significant burden on the health care system [1]. 

Plaque development and vulnerability to rupture are key processes in atherosclerosis, which lead to critical cardiovascular events through formation of thrombi. In view of the established finding that progression of vulnerable plaques results from interactions of factors involved in inflammation and lipid metabolism in arterial walls [1,2,3,4], suppression of plaque vulnerability is a primary therapeutic strategy.

Currently, reduction of low-density lipoprotein levels with hydroxymethylglutaryl coenzyme A reductase inhibitors (also known as statins) is a major therapeutic standard for atherosclerosis with known clinical benefits [5,6]. However, residual cardiovascular risk substantially remains in patients following statin treatment [7]. For instance, Cannon et al. [5] reported that more than 20% patients treated with high-dose statins present a recurrent event within 30 months after the onset of acute coronary syndrome. Thus, statins alone are insufficient to effectively treat atherosclerosis and further development of novel therapeutic agents remains an urgent medical requirement [8]. 

Accumulating evidence has demonstrated pivotal roles of matrix metalloproteinase-9 (MMP-9) mainly derived from infiltrating monocytes/macrophages in exacerbating plaque vulnerability [9,10,11,12]. MMP-9 degrades the extracellular matrix (ECM) including the protective fibrous cap of plaque, rendering plaques more prone to rupture [9,10,11]. Furthermore, MMP-9 facilitates monocyte/macrophage infiltration into plaques through ECM degradation and chemokine modulation, which contributes to increased burden of inflammation, thereby promoting a cascade of events leading to plaque rupture [11,12]. Several studies have suggested that MMP-9 is upregulated by the extracellular MMP inducer EMMPRIN, also known as cluster of differentiation 147 (CD147), which is located on leukocytes and vascular smooth muscle cells in atherosclerotic plaques [13,14,15]. CD147 is a cell surface glycoprotein that induces MMP-9 production through interactions with its ligand cyclophilin A (CypA) [16,17,18]. Therefore, interactions between CD147 and CypA present a potential therapeutic target to prevent plaque rupture.

**Figure 1:**
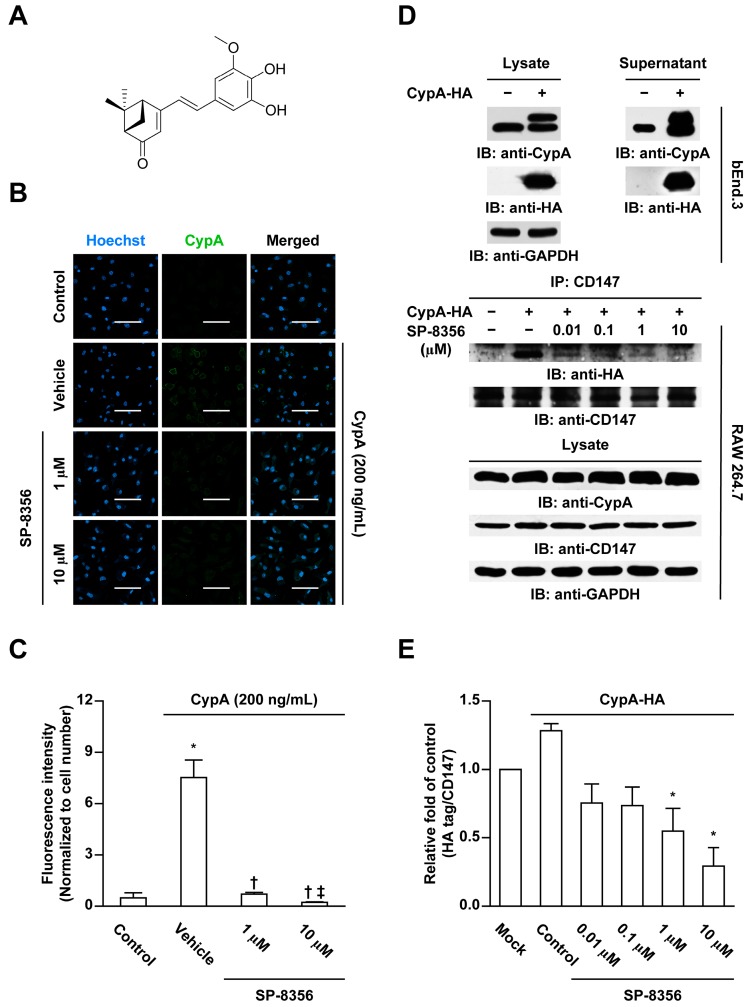
SP-8356 inhibits CD147-Cyclophilin A (CypA) interactions. (**A**) Structure of SP-8356; (**B**) representative images of immunocytochemial analysis of CypA binding to the cell membrane and (**C**) quantitative analysis of amount of binding CypA to the cell membrane. Macrophages were incubated with CypA in the absence or presence of SP-8356. Cell nuclei were counterstained with Hoechst 33342. Scale bar, 50 µm, magnification, 400×. Data are presented as means ± standard deviation (SD) of three independent experiments (* *p* < 0.05 vs. control. † *p* < 0.05 vs. vehicle. ‡ *p* < 0.05 vs. SP-8356 1 µM.); (**D**) representative images of immunoprecipitation (IP) analysis to assess inhibition of CD147-CypA interactions by SP-8356 and (**E**) quantitative analysis of amount of hemagglutinin (HA)-tagged CypA which was bound to CD147. Original gels are available in the Supplementary file. CypA-HA, HA-tagged CypA; IB, immunoblotting; bEnd.3, mouse brain endothelial cell line; RAW 264.7, mouse macrophage cell line; GAPDH, glyceraldehyde 3-phosphate dehydrogenase. Data are presented as means ± SD of three independent experiments. * *p* < 0.05 vs. control.

SP-8356((1S,5R)-4-(3,4-dihydroxy-5-methoxystyryl)-6,6-dimethylbicyclo[3.1.1]hept-3-en-2-one), a novel synthetic small-molecule drug, is a (1S)-(−)-verbenone derivative with anti-inflammatory and anti-oxidative activities (Figure 1A) [19]. In addition, we recently found that SP-8356 binds to CD147 and reduces neointimal hyperplasia through inhibition of MMP-9 activity [20]. We also found that SP-8356 has an anti-tumor effect through inhibition of CD147/MMP-9 pathway [21]. In the present study, we further examined whether binding of SP-8356 to CD147 can disrupt its interaction with CypA with consequent suppressive effects on plaque progression. The pharmacological efficacy of SP-8356 on advanced plaques and underlying mechanisms were explored using apolipoprotein E-deficient (ApoE KO) mice.

## 2. Results

### 2.1. SP-8356 Inhibits CD147-CypA Interaction

At first, we examined whether SP-8356 could interfere the CypA binding with cell membrane in rat monocyte-derived macrophages isolated from Sprague-Dawley (SD) rats. In immunocytochemical analyses, addition of CypA to macrophages led to an increase in CypA binding to the cell membrane and SP-8356 treatment completely suppressed CypA binding to the cell membrane (Figure 1B,C). As CD147 functions as a cell membrane receptor for CypA [16,17,18], to further characterize the underlying mechanism, we performed immunoprecipitation analyses to assess the direct effects of SP-8356 on CD147-CypA interactions. CypA is mainly present in the intracellular form but displays increased secretion during inflammatory reactions [18]. Secreted extracellular CypA is functionally important in CD147 interactions. However, during conventional immunoprecipitation, the lysis process makes it impossible to differentiate extra- from intracellular CypA. Accordingly, we used the transfection method to obtain secreted extracellular hemagglutinin (HA)-tagged CypA for treatment of mouse macrophage cell line (RAW 264.7) cells. As shown in Figure 1D, secreted HA-tagged CypA was successfully acquired in the supernatant. SP-8356 significantly suppressed CD147-CypA interactions in HA-tagged CypA-treated RAW 264.7 cells in a dose-dependent manner (Figure 1D,E).

### 2.2. SP-8356 Reduces CypA-Induced MMP-9 Activation and Monocyte Adhesion

**Figure 2:**
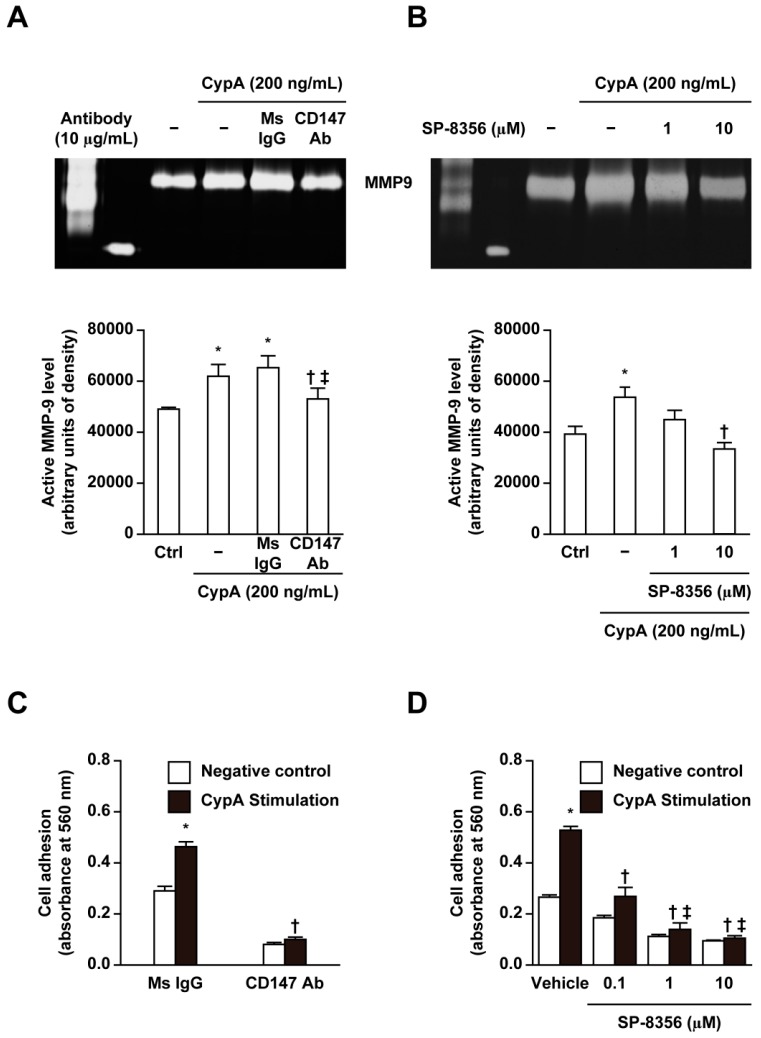
SP-8356 attenuates CypA-stimulated matrix metalloproteinase-9 (MMP-9) activation and monocyte adhesion. Rat monocyte-derived macrophages were treated with CypA in the absence and presence of mouse (Ms) IgG or anti-CD147 antibody (Ab) (**A**) or SP-8356 (**B**). Ctrl, Control. Data are presented as means ± SD of three independent experiments (* *p* < 0.05 vs. control. † *p* < 0.05 vs. vehicle. ‡ *p* < 0.05 vs. Ms IgG). Monocyte adhesion was quantified in the anti-CD147 Ab-treated (**C**) and SP-8356 treated groups (**D**). Data are presented as means ± SD of three independent experiments. In (C), * *p* < 0.05 vs. negative control without CypA stimulation; † *p* < 0.05 vs. Ms IgG with CypA stimulation. In (D), * *p* < 0.05 vs. negative control without CypA stimulation; † *p* < 0.05 vs. vehicle with CypA stimulation; ‡ *p* < 0.05 vs. SP-8356 0.1 µM.

Since binding of CypA to CD147 promotes MMP-9 activation and leukocyte adhesion [18], we examined the effects of SP-8356 on these processes. MMP activation by CypA was completely inhibited by the functional CD147 antibody (Figure 2A). Similarly, SP-8356 induced a significant reduction in MMP-9 activation in CypA-treated rat monocyte-derived macrophages (Figure 2B). Analogous to the functional CD147 antibody (Figure 2C), SP-8356 significantly attenuated CypA-induced leukocyte adhesion (Figure 2D).

### 2.3. SP-8356 Prevents the Formation of Plaque and Attenuates Its Vulnerability

**Figure 3:**
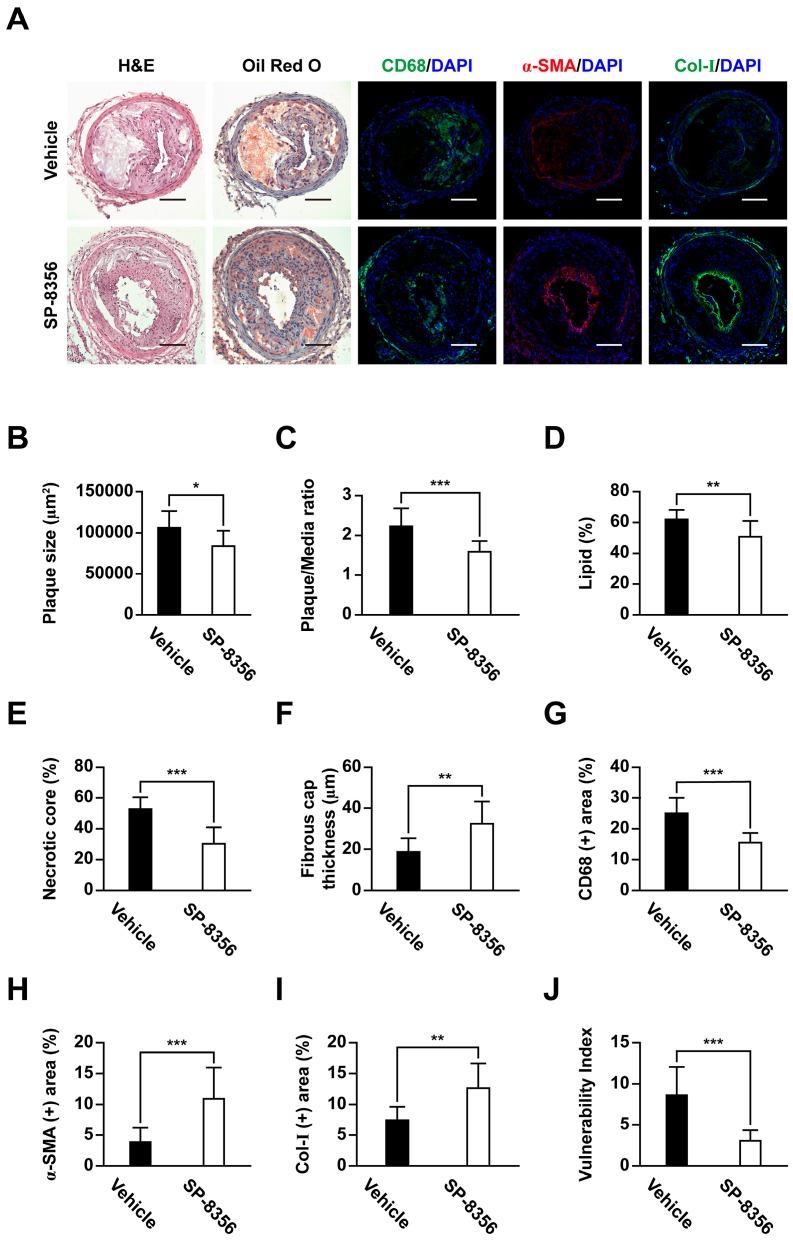
SP-8356 inhibits plaque development and improves plaque stability in ApoE KO mice. (**A**) representative images of cross-sectional carotid artery plaques (H&E; Hematoxylin and eosin staining, α-SMA; α-Smooth muscle-actin, Col-I; Collagen type I). Nuclei were counterstained with 4′,6-diamidino-2-phenylindole (DAPI). Scale bar, 100 µm (magnification, 100×); (**B**,**C**) quantitative analysis of plaque development in the cross-sectional plaque area (*n* = 12 for vehicle, *n* = 10 for SP-8356); (**D**–**J**) quantitative analysis of plaque vulnerability in the cross-sectional plaque area (*n* = 12 for vehicle, *n* = 10 for SP-8356). Data are presented as means ± SD (* *p* < 0.05, ** *p* < 0.01, *** *p* < 0.001).

Advanced plaque lesions successfully developed after partial ligation of carotid artery in ApoE KO mice (Figure 3A). Notably, SP-8356 induced a significant reduction in plaque size and plaque/media ratio (Figure 3A–C) with decreased lipid and necrotic core areas (Figure 3A,D,E). Conversely, fibrous cap thickness was considerably greater in the SP-8356-treated group (Figure 3A,F).

**Figure 4:**
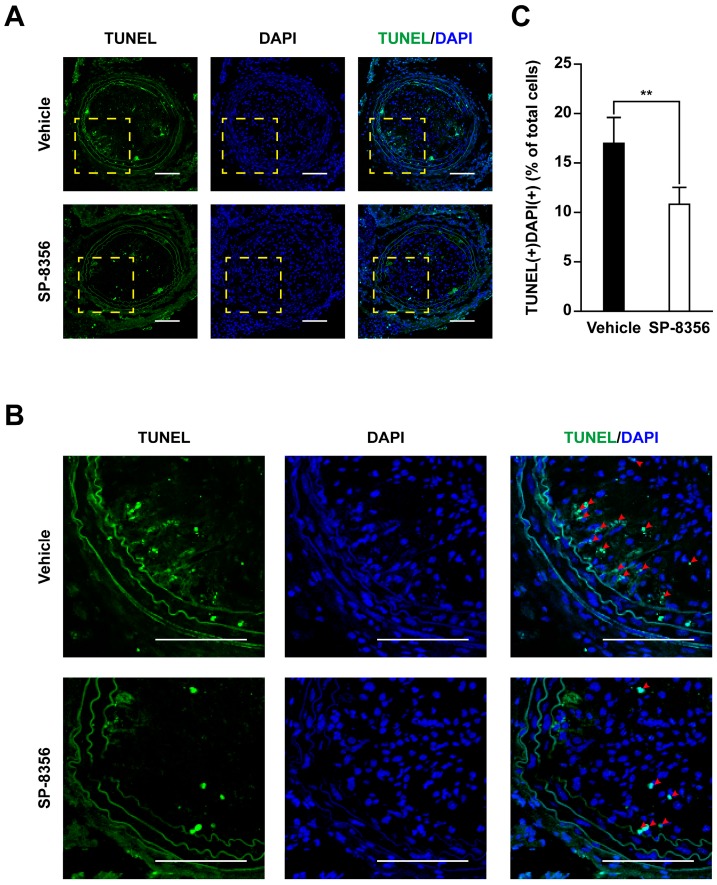
Effect of SP-8356 on cellular apoptosis in atherosclerotic plaques. (**A**) representative images of terminal deoxynucleotidyl transferase dUTP nick end labeling (TUNEL); (**B**,**C**) co-localization of quantitative analysis of TUNEL staining (green) and DAPI (blue) to measure cellular apoptosis in carotid plaques (*n* = 7 for vehicle, *n* = 6 for SP-8356). Co-localized cells (red arrows). Nuclei were counterstained with DAPI. Data are presented as means ± SD (scale bar 100 µm; magnification 100×, ** *p* < 0.01). Yellow box is the magnification 400×.

The relative contents of macrophages in plaques, assessed as the CD68-positive area, were markedly lower in the SP-8356-treated group (Figure 3A,G). In parallel, vascular smooth muscle cell (VSMC) proportion, as assessed by measurement of α-smooth muscle-actin (α-SMA) expression, and collagen type I expression within the plaque were increased in an SP-8356-treated group (Figure 3A,H,I). Accordingly, the plaque vulnerability index based on the ratio of lipid macrophage components (lipid area + macrophages) and fibromuscular component (VSMC + collagen) [22] was significantly lower in the SP-8356-treated group (Figure 3J). Furthermore, SP-8356 decreased the number of terminal deoxynucleotidyl transferase dUTP nick end labeling (TUNEL)-positive apoptotic cells in plaque lesions (Figure 4).

No significant differences between vehicle- and SP-8356-treated groups were observed in terms of body weight, serum total cholesterol, and serum triglyceride (Figure S1). 

### 2.4. SP-8356 Suppresses Collagenase Activity, MMP-9, and CypA within Plaque Lesions

**Figure 5:**
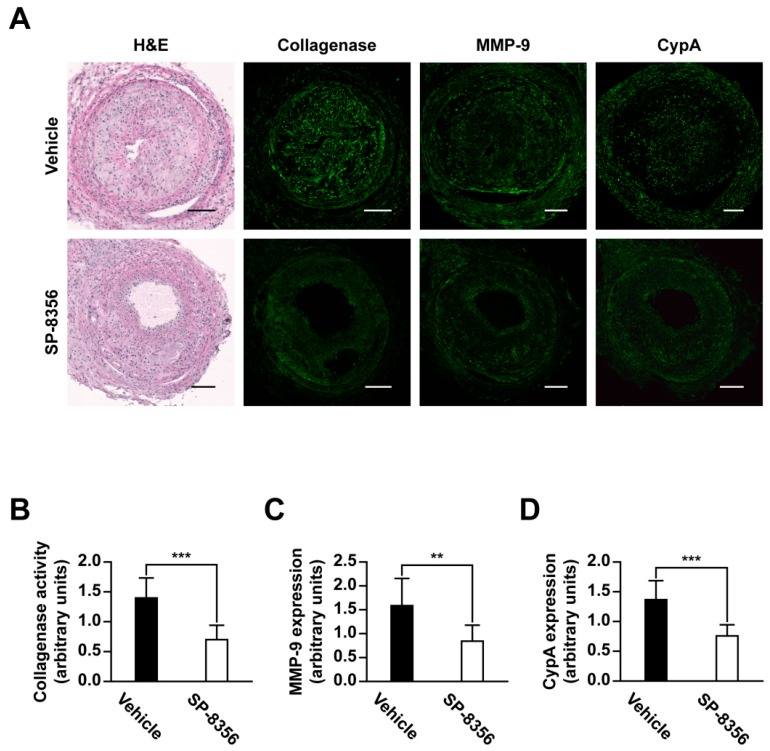
Effects of SP-8356 on MMP activation, MMP-9 and CypA expression in atherosclerotic plaques. (**A**–**D**) representative in situ zymography, immunohistochemical staining, and quantitative analysis of plaques (*n* = 12 for vehicle, *n* = 10 for SP-8356) (scale bar 100 µm; magnification 100×). Data are presented as means ± SD (** *p* < 0.01, *** *p* < 0.001).

Several studies have established that overexpression of MMP-9 promotes plaque destabilization [9,10,11,12]. Accordingly, we examined whether SP-8356 affects MMP-9 activation and expression in plaque lesions. In situ zymography data revealed a decrease in collagenase activity in SP-8356-treated mice (Figure 5A,B), along with reduced MMP-9 (Figure 5A,C) and CypA expression in plaque lesions (Figure 5A,D).

## 3. Discussion

Changes in plaque composition leading to an increase in the lipid-rich necrotic core covered by a thin fibrous cap are essential for plaque destabilization resulting in rupture. Thin fibrous caps typically comprise abundant macrophages and a small number of VSMCs [23,24,25]. These deleterious changes are mediated by various inflammatory factors, among which MMP has been identified as the most important coordinator [26]. In the present study, we characterized a novel synthetic drug, SP-8356, that effectively suppressed plaque composition changes, inflammatory responses, and apoptotic progression.

MMP is the most critical factor responsible for plaque rupture [26]. However, broad-spectrum MMP inhibitors have failed to show therapeutic efficacy in preclinical experimental models of atherosclerosis [27,28]. MMPs play important roles in development, injury, and repair in pathophysiological conditions [29], which may be the underlying reason for adverse effects, such as musculoskeletal pain, inflammation, and other complications, associated with MMP inhibitors in clinical studies that are not evident in preclinical studies [30,31].

A long history of failed clinical trials using broad-spectrum MMP inhibitors has spurred research on the development of selective inhibitors for individual MMPs. Lutten et al. [32] reported that genetic deletion of MMP-9 inhibits atherosclerotic plaque lesion progression in ApoE KO mice. Furthermore, overactivation of MMP-9 in plaque lesion is known to be associated with accelerated plaque rupture [9,11]. Among the MMP family members, MMP-9 has been identified as an effective therapeutic target for improving plaque vulnerability. Recent research has thus focused in the development of selective rather than broad-spectrum MMP inhibitors, which may be more successful for therapy [33].

CD147 is a key player in the inflammation process [18,34,35]. Specific inhibition of CD147 with small interfering (si) RNA or monoclonal antibody (mAb) has been shown to exert profound anti-atherosclerotic effects, both in vitro and in vivo, through inhibition of CD147-mediated MMP-9 induction [34,36]. Data from the present study showed that SP-8356 suppresses the CD147-mediated MMP pathway rather than directly inhibiting MMP-9. To our knowledge, SP-8356 is the first novel small-molecular compound identified that appears to exert anti-atherosclerotic effects through effects on the CD147/MMP-9 pathway. Regulation of MMP activities via CD147 inhibition could present an alternative to overcome the toxicities of direct MMP inhibitors. Given that CD147 is a drug target, mAb therapy can also be considered. However, despite developments in mAb therapy, several limitations continue to impede their utility, such as high production cost, unfavorable pharmacokinetics and unclear mode of action [37]. In contrast, SP-8356 is easy to manufacture, low-cost and offers the significant advantage of oral bioavailability, similar to other small-molecular drugs.

CD147-evoked promotion of the inflammation cascade occurs through interactions with several ligands, including CypA and intergrins [35]. Previous studies have shown that interactions of CD147 with CypA recruit inflammatory cells to inflamed tissue with upregulation of MMP production, and are closely associated with cardiovascular diseases [18,38]. In addition to CD147, its ligand CypA is regarded as a potential therapeutic target in atherosclerosis. However, the overall therapeutic effect on atherosclerosis via inhibition of CypA is a subject of controversy. Genetic ablation of CypA in ApoE KO mice has been shown to be anti-atherogenic [39], whereas pharmacological inhibition of CypA in ApoE KO mice promotes atherosclerosis [40]. Further research is warranted to elucidate the association between CypA inhibition and anti-atherosclerotic effects.

CD147-CypA interaction can promote nuclear factor kappa B (NF-κB) nuclear translocation thereby upregulating leukocyte adhesion and migration, which eventually leads to accelerating plaque progression [18]. In our previous study, we found that SP-8356 binds to CD147 and inhibits NF-κB nuclear translocation thereby limiting the proliferation rate of breast cancer cells [21]. Thus, the inhibitory effect of SP-8356 on CD147-CypA interaction-evoked NF-κB nuclear translocation may be at least in part involved in the anti-atherosclerotic effect of SP-8356.

In the present study, SP-8356 suppressed the CypA level in atherosclerotic plaques of ApoE KO mice to a significant extent (Figure 5A,D). Secretion of CypA is upregulated by inflammatory stimuli [18] and reactive oxygen species [41]. Accordingly, we propose that the anti-inflammatory and anti-oxidative activities of SP-8356 reported by our group [19,21] are associated with reduction of the CypA level in atheroaclerotic plaques. Recently, elevated CypA levels were reported to have a prognostic impact on all-cause death, coronary revascularization and rehospitalization of patients with coronary artery disease [42]. Furthermore, CypA offers superior prognostic value to other conventional biomarkers, such as high-sensitivity C-reactive protein and brain natriuretic peptide [42]. The collective findings support that the feasibility of utilizing the CypA level as a prognostic biomarker and its reduction by inhibitors, such as SP-8356, leading to prognostic improvements in patients with atherosclerosis-related clinical events.

CD147 homophilic interactions, such as dimerization and CD147-CypA interactions, are the two dominant mechanisms leading to MMP-9 upregulation [16]. Accumulated evidence from our previous [20] and current studies suggests that SP-8356 can synergistically inhibit MMP-9 activity by binding to CD147 and simultaneously attenuating CD147 dimerization and CD147-CypA interactions. However, detailed basic mechanisms require further investigation.

Although SP-8356 exerts anti-atherosclerotic effects in ApoE KO mice, the most popular murine model of atherosclerosis, physiological differences between our experimental animal model and humans, such as cholesterol metabolism, may limit its efficacy as a clinical agent [43], and extrapolation of data to clinical human practice should therefore be made with caution. Nevertheless, while further research is required to establish the detailed mechanisms underlying its anti-atherosclerotic effects, SP-8356 clearly offers promise as a novel drug candidate for atherosclerotic plaque formation and vulnerability. From a clinical point of view, it is also expected that SP-8356 can be used synergistically with conventional cholesterol-lowering therapies such as statins. 

## 4. Materials and Methods

### 4.1. Cell Culture

The mouse brain endothelial cell line bEnd.3 (ATCC CRL-2299) was purchased from American Type Culture Collection (ATCC, Manassas, VA, USA) and the mouse macrophage cell line, RAW 264.7, kindly provided from by Professor Namhyun Jung from Korea University. All cells were cultured in Dulbecco’s modified eagle medium (DMEM; Welgene, Daegu, Korea) supplemented with 10% fetal bovine serum (FBS; HyClone, GE Healthcare Life Sciences, Logan, UT, USA) and 1% penicillin/streptomycin (HyClone, GE Healthcare Life Sciences, Logan, UT, USA) at 37 °C in a 5% CO_2_ humidified atmosphere.

Rat peripheral blood mononuclear cells were isolated from heparinized blood obtained from normal Sprague–Dawley (SD) rats via density gradient centrifugation (Ficoll-Plaque Plus, GE Health Care Life Sciences, Pittsburgh, PA, USA) according to the manufacturer’s instruction. Differentiation into macrophages was induced by a recombinant rat macrophage colony-stimulating factor (15 ng/mL; Peprotech, Rocky Hill, NJ, USA) with 1% penicillin/streptomycin (Hyclone, GE Healthcare Life Sciences, Logan, UT, USA) and 10% heat-inactivated FBS (HyClone, GE Healthcare Life Sciences, Logan, UT, USA) for 7 days.

### 4.2. Immunocytochemistry

To assess whether SP-8356 affects binding of CypA to the cell surface, rat monocyte-derived macrophages isolated from SD rats were cultured onto cover glasses in 24-well plates for 7 days. Cells were pre-treated with different concentration of SP-8356 for 30 min and subsequently treated with recombinant CypA (200 ng/mL; ab86219, Abcam, Cambridge, MA, USA) without permeabilization. Cells were washed with phosphate-buffered saline (PBS) and blocked with a 10% goat serum, followed by incubation with a rabbit polyclonal anti-CypA antibody (ab41684, Abcam, Cambridge, MA, USA) for 30 min at 4 °C and goat anti-rabbit Alexa Fluor 488 (A11034, Invitrogen, Carlsbad, CA, USA) as the secondary antibody. Nuclei were counterstained with Hoechst 33342 solution.

### 4.3. Immunoprecipitation

Immunoprecipitation experiments were conducted to examine whether SP-8356 inhibits CD147-CypA interactions. To this end, we used a commercially available human CypA coding sequence with a pCMV-C-HA vector (HG10436-CY, Sino Biological Inc., Beijing, China). Briefly, bEnd.3 cells were transfected with individual plasmids using Lipofectamine 3000 (Invitrogen, Carlsbad, CA, USA) for 48 h. To release HA-tagged CypA into extracellular medium, transfected cells were exposed to oxygen-glucose deprivation (OGD) for 6 h and the culture supernatants collected. RAW 264.7 cells were co-treated with the collected supernatant fractions and different concentrations of SP-8356 for 30 min at 37 °C, harvested and lysed with lysis buffer containing a protease inhibitor cocktail for 60 min on ice. Lysates were centrifuged at 15,000 rpm for 15 min at 4 °C and the supernatant fractions incubated with monoclonal anti-CD147 antibody (sc46700, Santa Cruz Biotechnology, Santa Cruz, CA, USA) at 4 °C overnight. Next, supernatants were added to pre-washed protein A Sepharose beads (17-5280-01, GE Healthcare, Munich, Germany) and incubated for 2 h at 4 °C. Beads were rewashed and bound proteins eluted by boiling in sodium dodecyl sulfate (SDS) sample buffer, followed by separation via 15% SDS-polyacrylamide gel electrophoresis (PAGE). To examine protein expression, clarified lysates were electrophoresed, transferred onto nitrocellulose membrane, probed with anti-HA (ab18181, Abcam, Cambridge, MA, USA) and anti-CD147 (ab108317, Abcam, Cambridge, MA, USA) and detected using an electrochemiluminescence (ECL) assay kit (GE Healthcare Life Sciences, Pittsburgh, PA, USA).

### 4.4. Gelatin Zymography

MMP activities of macrophages were evaluated via gelatin zymography. Monocyte-derived macrophages isolated from SD rats were treated with SP-8356 on the presence of CypA (200 ng/nl; ab86219, Abcam, Cambridge, MA, USA), functional CD147 antibody (ab119114, Abcam, Cambridge, MA, USA), or mock mouse IgG antibody (sc2025, Santa Cruz Biotechnology, Santa Cruz, CA, USA) for 24 h. Conditioned media were collected, centrifuged to remove cellular debris and concentrated with a Microcon centrifugal filter device (Millipore, Billerica, MA, USA). Samples were mixed with non-reducing loading buffer without heating and loaded onto a 10% SDS gel containing 1 mg/mL gelatin (JT Baker Chemical Co., Phillipsburg, NJ, USA). Proteins were separated via electrophoresis at 125V for 90 min. An MMP-9 recombinant protein (ab168863, Abcam, Cambridge, MA, USA) was loaded as a positive control. Following electrophoresis, gels were rinsed twice for 30 min in Novex zymography renaturing buffer (Invitrogen, Carlsbad, CA, USA) and subsequently incubated with zymogram developing buffer (Invitrogen, Carlsbad, CA, USA). After overnight reaction, the gel was stained with Simply Blue Safe Stain (Invitrogen, Carlsbad, CA, USA).

### 4.5. Adhesion Assay

Rat peripheral blood-derived monocytes (10^7^ cells/mL) were incubated with CypA and SP-8356 in 0.5% bovine serum albumin/Dulbecco’s PBS for 10 min at 37 °C. The mixture was distributed on a plate coated with a Matrigel matrix (356237, BD Biosciences, San Jose, CA, USA) for an additional 60 min at 37 °C. The plate was extensively washed to remove non-adherent cells and the remaining firmly attached cells were fixed with 4% paraformaldehyde for 20 min at 4 °C. Adherent cells were stained with 0.2% crystal violet (V5265, Sigma-Aldrich, St. Louis, MO, USA) in PBS for 10 min at room temperature (RT). Next, plates were washed with distilled water to remove unfixed crystal violet and dried in air, followed by lysis with 10% SDS for 10 min at RT. The absorbance of each well was measured with a SpectraMAX 190 microplate reader (Molecular Devices, Sunnyvale, CA, USA) at 560 nm. 

### 4.6. Animals

Male ApoE KO mice (7 weeks old, 20 g body weight) were purchased from Jackson Laboratory (C57BL/6J-ApoE, Bar Harbor, ME, USA) and maintained under 12 h/12 h day/night cycle, with water and meals provided ad libitum. All experimental protocols were approved by the Institutional Animal Care and Use Committee of Korea University College of Medicine (Approval No. KOREA-2017-0120; approval date 31/07/2017). 

### 4.7. Induction of Advanced Plaque

After 2 weeks of acclimation, right partial carotid artery ligation was conducted as described previously [44]. In brief, anesthesia was started with 3.5% isoflurane in a 2:1 N_2_O/O_2_ mixture in a vented anesthesia chamber and maintained by inhalation through a nasal cone of 2 to 2.5 isoflurane in a 2:1 N_2_O/O_2_ mixture. After disinfection with betadine, incision was made in the neck and right carotid artery (RCA) was exposed by blunt dissection. Three caudal branches of RCA (i.e., right external carotid, internal carotid, and occipital artery), except superior thyroid artery, were ligated with 6-0 silk sutures. All mice were fed daily with commercially available Paigen’s high-fat diet (D12336, Research Diets, NJ, USA) for 3 weeks. 

### 4.8. Drug Treatment

Mice were subdivided into vehicle and SP-8356 (50 mg/kg) groups. SP-8356 was added to drinking water from 1 day to 3 weeks after ligation of carotid artery. The drug dose was determined on the basis of average daily water consumption (6 mL/day for each mouse) [45] and body weights, which were measured every day.

### 4.9. Blood Analysis

Blood samples were collected immediately before sacrifice. Total cholesterol and triglyceride levels in serum were measured using a FUJI DRI-Chemiclinical Chemistry Analyzer (FUJI DRI-CHEM 4000i, Fuji Film, Tokyo, Japan).

### 4.10. Histopathology

Mice were euthanized via carbon dioxide inhalation after 3 weeks of drug administration. Harvested common carotid arteries were fixed with 4% paraformaldehyde and preserved in 30% sucrose solution. Next, tissue was embedded in optimal cutting temperature compound (Scigen Scientific, Gardena, CA, USA). Axial sections of 4 µm thickness were cut with a cryostat microtome (Leica CM 3050 S, Leica Microsystems, Wetzlar, Germany). Serial sections were obtained down stream of bifurcation of external and internal carotid arteries, stained with hematoxylin and eosin for plaque morphology and oil red O for lipid deposition, and evaluated using an upright light microscope (BX51, Olympus, Tokyo, Japan). The lesion areas of each serial section were measured using ImageJ (version 1.45s, Bethesda, MD, USA). For the quantitation of lipid deposition, region of interest (ROI) was manually drawn on plaque area and the areas with positive oil red O stained lesions were measured using ImageJ and divided by plaque area. In accordance with the guidelines for experimental atherosclerosis studies by the American Heart Association [46], lesions were analyzed in a blinded manner and quantified as an average of 6 sections, each 100 µm apart from each other.

### 4.11. Immunohistochemistry

Sections were incubated with following antibodies: anti-α-SMA (ab7817, Abcam, Cambridge, MA, USA), anti-CD68 (ab125212, Abcam, Cambridge, MA, USA), anti-collagen type I (ab34710, Abcam, Cambridge, MA, USA), and anti-MMP-9 (AB19016, Merck Millipore, Billerica, MA) and anti-CypA (ab41684, Abcam, Cambridge, MA, USA). Alexa Fluor 555-conjugated goat anti-mouse IgG (A21424, Carlsbad, CA, USA) and Alexa Fluor 488-conjugated goat anti-rabbit IgG (A11034, Carlsbad, CA, USA) were used as secondary antibodies. Nuclei were counterstained with 4′, 6-diamidino-2-phenylindole (DAPI). All images were acquired using a confocal microscope (LSM800, Carl Zeiss, Oberkochen, Germany). For the quantitative analysis of α-SMA, CD68, and collagen type I expression, ROI was manually drawn on plaque area and the areas with positively stained lesions were measured using ImageJ and divided by plaque area. For the quantitative analysis of MMP-9 and CypA expression, ROI was manually drawn on plaque area and the fluorescence intensity of each pixel in ROI was measured using ImageJ and normalized to the plaque area. 

### 4.12. Analysis of Collagenase Activity

To evaluate the collagenase activity of plaques, we performed in situ zymography. Harvested fresh tissue was frozen immediately without fixation. Freshly cut 4 µm thick sections were incubated with a green-fluorescence labeled gelatin substrate (DQ gelatin, Molecular Probes, Carlsbad, CA, USA) according to the manufacturer’s instructions, and collagenase activity analyzed under a confocal microscope (LSM800, Carl Zeiss, Oberkochen, Germany). For the quantitative analysis of collagenase activity, ROI was manually drawn on plaque area and the fluorescence intensity of each pixel in ROI was measured using ImageJ and normalized to the plaque area. 

### 4.13. In Situ Detection of Apoptotic Cells

Apoptotic cells in plaque cryosections were determined using a (TUNEL in situ apoptosis detection kit (Dead-End Colorimetric TUNEL system, Promega, Madison, WI, USA) according to the manufacturer’s instructions. Sections were counterstained with DAPI. The number of TUNEL-positive cells was normalized to the total number of DAPI-positive cells in the lesions. 

### 4.14. Statistical Analysis

All data were presented as means ± standard deviation. The Shapiro–Wilk test was used to assess normal distribution of variables. For parametric analysis, one-way analysis of variance (ANOVA) followed by a post-hoc Tukey’s test was used for multiple comparisons and a Student’s *t*-test applied for comparison between groups. For non-parametric analysis, the Kruskal–Wallis test with post-hoc Conover test was used for multiple comparison data and a Mann–Whitney *U* test was used to compare the values between the two groups. SPSS version 17.0 (SPSS Inc., Chicago, IL, USA) and MedCalc version 18.5 (MedCalc, Mariakerke, Belgium) were employed for data analysis, with data considered statistically significant at *p*-values < 0.05.

## Supplementary Materials

Supplementary materials can be found at https://www.mdpi.com/1422-0067/21/1/95/s1. Figure S1: Body weights (A) and blood lipid profiles (B, C) of vehicle- and SP-8356 treated ApoE KO mice (*n* = 12 for vehicle, *n* = 10 for SP-8356) (TC; Total cholesterol, TG; triglyceride, *n. s*.; not significant).
