# Supplementary material for: SP-8356, a Novel Inhibitor of CD147-Cyclophilin A Interactions, Reduces Plaque Progression and Stabilizes Vulnerable Plaques in apoE-Deficient Mice
Source: Int J Mol Sci. 2019 Dec 21;21(1):95. doi: 10.3390/ijms21010095 (PMC6981359; doi:10.3390/ijms21010095)
Supplement: Supplementary file 1 [file ijms-21-00095-s001.pdf]

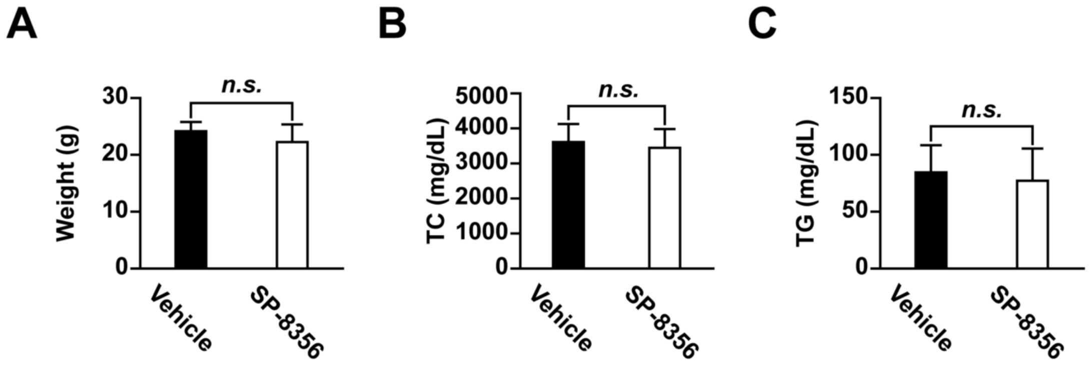

Figure 1. Body weights (**A**) and blood lipid profiles (**B**, **C**) of vehicle- and SP-8356 treated ApoE KO mice ($n = 12$ for vehicle, $n = 10$ for SP-8356) (TC; Total cholesterol, TG; triglyceride, *n. s.*; not significant).
